# Enhanced Sensitivity of a Love Wave-Based Methane Gas Sensor Incorporating a Cryptophane-A Thin Film

**DOI:** 10.3390/s18103247

**Published:** 2018-09-27

**Authors:** Wen Wang, Shuyao Fan, Yong Liang, Shitang He, Yong Pan, Caihong Zhang, Chuan Dong

**Affiliations:** 1 Institute of Acoustics, Chinese Academy of Sciences, Beijing 100190, China; fanshuyao@mail.ioa.ac.cn (S.F.); liangyong@mail.ioa.ac.cn (Y.L.); heshitang@mail.ioa.ac.cn (S.H.); 2 University of Chinese Academy of Sciences, Beijing 100190, China; 3 State Key Laboratory of NBC Protection for Civilian, Yangfang, Changping District, Beijing 102205, China; 4 School of Chemistry and Chemical Engineering, Shanxi University, Taiyuan 030006, China; chzhang@sxu.edu.cn (C.Z.); dc@sxu.edu.cn (C.D.)

**Keywords:** cryptophane A, differential oscillation, Love wave methane gas sensor, waveguide effect, self-temperature compensation

## Abstract

A Love wave-based sensing chip incorporating a supramolecular cryptophane A (CrypA) thin film was proposed for methane gas sensing in this work. The waveguide effect in the structure of SiO_2_/36° YX LiTaO_3_ will confine the acoustic wave energy in SiO_2_ thin-film, which contributes well to improvement of the mass loading sensitivity. The CrypA synthesized from vanillyl alcohol by a double trimerisation method was dropped onto the wave propagation path of the sensing device, and the adsorption to methane gas molecules by supramolecular interactions in CrypA modulates the acoustic wave propagation, and the corresponding frequency shifts were connected as the sensing signal. A theoretical analysis was performed to extract the coupling of modes for sensing devices simulation. Also, the temperature self-compensation of the Love wave devices was also achieved by using reverse polarity of the temperature coefficient in each media in the waveguide structure. The developed CrypA coated Love wave sensing device was connected into the differential oscillation loop, and the corresponding gas sensitive characterization was investigated. High sensitivity, fast response, and excellent temperature stability were successfully achieved.

## 1. Introduction

**Figure 1:**
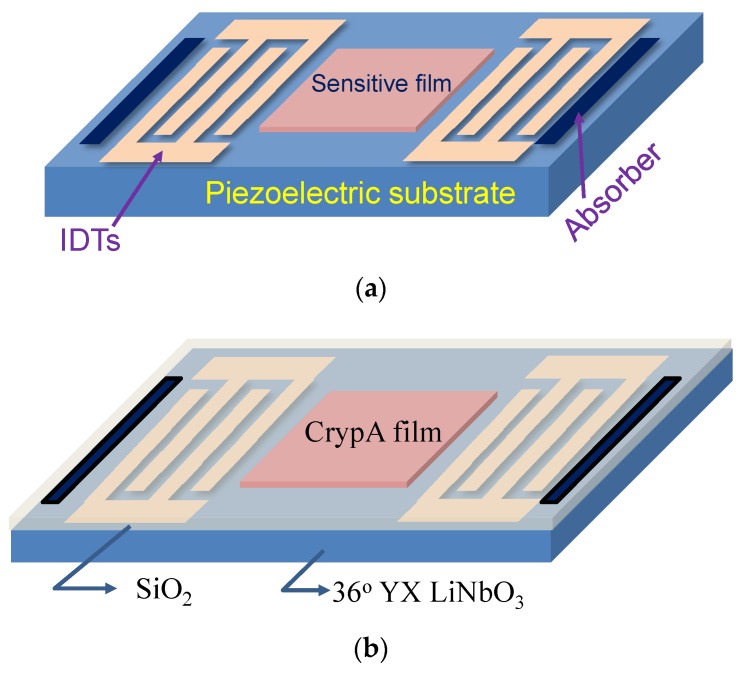
The schematic of SAW based gas sensor (**a**) and Love wave based sensor (**b**).

**Figure 2:**
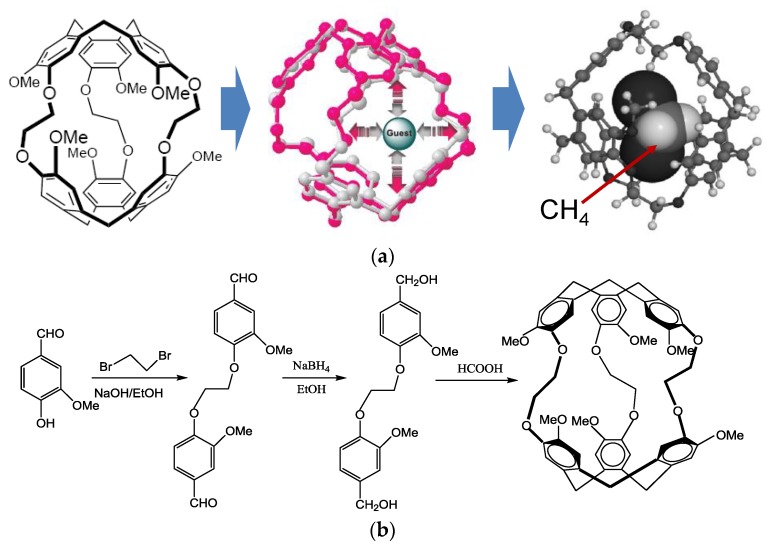
(**a**) supramolecular interactions of CrypA towards CH_4_ and (**b**) synthesis of cryptophane-A.

Underground mine methane gas poisoning or explosions can cause huge casualties and property losses. Methane gas (CH_4_) is a colorless, odorless and flammable gas with a lower explosive limit (LEL) concentration of about 5% by volume in air. Therefore, establishment of a rapid and sensitive methane gas monitoring system should be an effective way to respond to such issues. Among the available approaches for sensing H_2_, NO_2_, SO_2_, H_2_S and various other chemical agents surface acoustic wave (SAW) sensors exhibit some unique advantages such as low cost, fast response and high sensitivity, which have been reported [1,2,3,4,5,6] since the pioneering work of Wohltjen [7]. A typical SAW-based gas sensor is composed of the SAW device and the sensitive interface on top of it. A schematic of a SAW gas sensor is depicted in Figure 1a. The selective adsorption in the sensitive material modulates the SAW propagation along the SAW device, and the corresponding frequency shift of the sensing device is collected using a differential oscillator. Recently, a methane-sensitive material named as cryptophane A (CrypA) has attracted great interest because of its excellent selectivity for methane gas [8,9,10], and its gas adsorption behavior that can be described as a supramolecular interaction (Figure 2a) among the host and methane molecules arising from size complementarity and efficient van der Waals interactions, with amazing affinity towards methane gas (CH_4_) that induces excellent selectivity and sensitivity. A typical synthesis procedure of CrypA is described in Figure 2b, which follows a two-step method [11]. Some meaningful results were observed with a CrypA coated quartz crystal microbalance (QCM device) or SAW devices [10,12,13]. An outstanding advantage of them is that the sensor works at room temperature, which is beneficial for reducing the system power and attractive in the underground mining environment.

Recently, so-called Love wave devices were explored for gas sensing because of the obvious improvement in mass sensitivity arising from the waveguide effect [14,15]. Typical Love wave devices are composed of a piezoelectric substrate supporting a shear horizontal (SH) SAW, and a thin-guiding layer on top of the piezoelectric substrate. Due to the waveguide effect, the SAW was confined into the thin-guiding layer, and it is more sensitive to surface mass perturbations. The sensitivity achieved from the Love wave sensing devices was 10 times higher than that of the typical Rayleigh surface acoustic wave (R-SAW) ones [16]. Another advantage of the Love wave mode for gas sensing is the temperature-compensation of the device itself by choosing proper guiding materials possessing reverse polarization of the temperature coefficient to the piezoelectric substrate [17,18]. Wang et al. proposed a temperature-compensated Love wave device using the waveguide structure of SiO_2_/36° YX LiTaO_3_, which corresponding Love wave characteristics including dispersion relation and temperature coefficient of frequency (TCF) were investigated theoretically by solving the coupled electromechanical field equation, and the optimal waveguide structure was determined. Hence, Love wave devices are becoming a research hotspot in gas sensing.

In this work, a temperature-compensated Love wave device for methane gas sensing was proposed, which is composed of a waveguide structure of SiO_2_/36° YX LiTaO_3_ and a CrypA thin-film on top of the SiO_2_ guiding layer, as shown in Figure 1b. The 36° YX LiTaO_3_ substrate offers a large piezoelectric coupling coefficient κ^2^ (5.6%) and higher shear velocity (4202 m/s) over the SiO_2_ guiding layer (2850 m/s), which is beneficial for reducing the acoustic attenuation and advances in mass sensitivity [18]. Also, the SiO_2_ guiding layer possesses opposite polarization of the temperature of coefficient (Tcf) against the 36° YX LiTaO_3_, hence, lower Tcf of the hybrid Love wave device is expected by varying the SiO_2_ thickness. A theoretical simulation using FEM analysis was performed to determine the coupling of modes (COM) parameters for the Love wave sensing device simulation. The Love wave devices were fabricated by using the standard photolithographic process and ion-assisted e-beam evaporation. The synthesized CrypA was solved in tetrahydrofuran (THF), and dropped onto the wave propagation path. The prepared Love wave sensing device was connected to a differential oscillation loop, and the mixed frequency signal against the reference device was collected by the frequency acquisition module made by FPGA. The proposed Love wave sensor was characterized at room temperature (25 °C), and the corresponding performance features such as sensitivity, temperature stability, detection limit, and repeatability, were studied experimentally.

## 2. COM Simulation for Love Wave Sensing Devices

**Figure 3:**
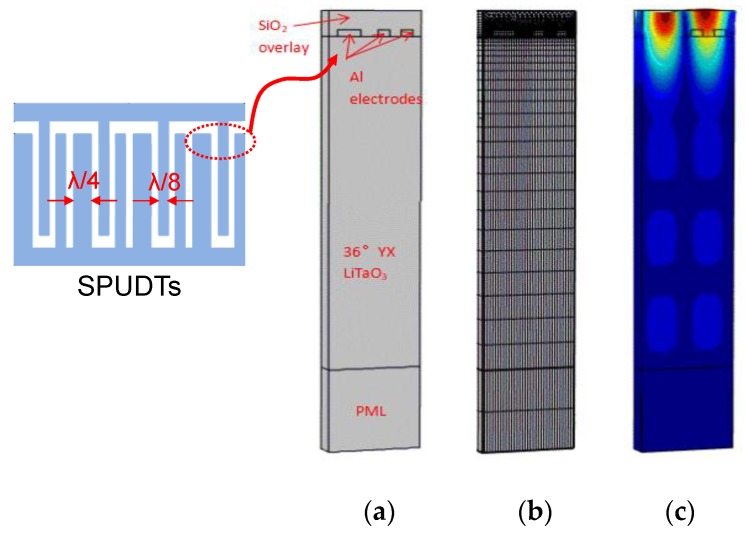
FEM analysis on Love wave devices (**a**) waveguide structure of SiO_2_/Al electrodes/36° YX LiTaO_3_; (**b**) Meshing structure and (**c**) Love wave displacement profile.

In this contribution, the FEM analysis was performed to describe the Love wave propagation in the waveguide structure of SiO_2_/Al electrodes/36° YX LiTaO_3_/PML (perfect match layer), as depicted in Figure 3. Here, single phase unidirectional transducers (SPUDTs) composed of interdigital electrodes with a width of λ/8, and an inserted reflection electrode with width of λ/4, were used to form the devices to reduce the insertion loss by controlling the wave propagation in one direction on the crystal surface [19]. The corresponding coupling of modes (COM) parameters defined by Equations (1)–(3) for device simulation can also be determined for Love wave sensing device simulation:

(1) $$\{\begin{array}{l} v=\lambda \frac{\left(f_{\mathrm{sc}+}+f_{\mathrm{sc}-}\right)}{2},\\ \left|\kappa \right|=\frac{2\pi }{\lambda }\frac{f_{\mathrm{sc}+}-f_{\mathrm{sc}-}}{f_{\mathrm{sc}+}+f_{\mathrm{sc}-}} \end{array}\;$$

(2) $$\{\begin{array}{l} \left|\alpha \right|=\sqrt{\frac{\omega C_{n}W\pi }{\lambda^{2}}\left(\frac{f_{\mathrm{oc}+}+f_{\mathrm{oc}-}}{f_{\mathrm{sc}+}+f_{\mathrm{sc}-}}-1\right)},\\ \cos\left(∠\alpha^{2}/\kappa \right)=\frac{{\left(f_{\mathrm{oc}+}-f_{\mathrm{oc}-}\right)}^{2}-{\left(f_{\mathrm{sc}+}-f_{\mathrm{sc}-}\right)}^{2}-{\left[\left(f_{\mathrm{oc}+}+f_{\mathrm{oc}-}\right)-\left(f_{\mathrm{sc}+}+f_{\mathrm{sc}-}\right)\right]}^{2}}{2\left(f_{\mathrm{sc}+}-f_{\mathrm{sc}-}\right)\left[\left(f_{\mathrm{oc}+}+f_{\mathrm{oc}-}\right)-\left(f_{\mathrm{sc}+}+f_{\mathrm{sc}-}\right)\right]} \end{array}\;$$

(3) $$C_{n}=\frac{W_{e}}{{\left(\Delta V\right)}^{2}W}\;$$

here, parameters of *v*, *κ*, *α* and *C* are the propagation velocity, coupling coefficient, excitation coefficient, and static capacitance. *f*_sc+_, *f*_sc−_, *f*_oc+_, and *f*_oc−_ denote the up and down boundary frequency of the stopband in periodic shorted grating and open grating. *W* and *λ* are the acoustic aperture and corresponding wavelength. Using the FEM method (COMSOL Multiphysics software) and the mechanical parameters of the 36° YX LiTaO_3_ piezoelectric substrate and SiO_2_ guiding layer [15], the modal analysis towards Love wave propagations in SiO_2_/SPUDTs/piezoelectric substrate can be well conducted, and corresponding SAW displacement profile in SPUDTs was calculated as depicted in Figure 2a. Following the modal analysis, the harmonic response analysis was performed to achieve the admittance characteristics, and the corresponding *f*_sc+_, *f*_sc−_, *f*_oc+_ and *f*_oc−_ can be extracted by searching the eigenfrequencies in normalized admittances, and allowing the extraction of COM parameters as SAW velocity, coupling coefficient and excitation coefficient. Moreover, a static analysis was performed to the structure of SPUDTs/piezoelectric substrate to obtain the static electric field energy, and hence, the static capacitance can also be determined by Equation (3).

**Figure 4:**
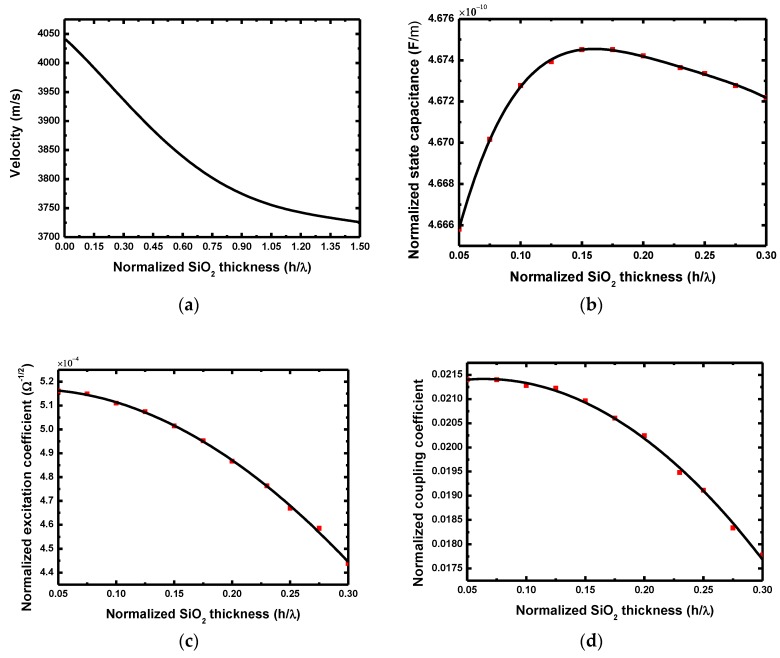
The calculated COM parameters varying with SiO_2_ thickness: (**a**) velocity; (**b**) static capacitance; (**c**) excitation coefficient and (**d**) coupling coefficient.

Obviously, the COM parameters vary with the SiO_2_ thicknesses, which are plotted in Figure 4. The SiO_2_ overlay slows down the wave propagation velocity (Figure 4a) because of the mass loading effect, that is, the acoustic wave velocity decreases with the increase of the SiO_2_, and close to the SH-velocity in SiO_2_ when larger thickness is applied. The variation trend of the static capacitance is different from the former, it appears that there exists a normalized SiO_2_ thickness to achieve a max static capacitance (Figure 4b). The excitation coefficient and coupling coefficient decreases with the thickness of the SiO_2_ overlay, as described in Figure 4c,d.

Moreover, there exists an optimum SiO_2_ thickness allowing self temperature-compensation and maximum mass loading sensitivity [17], and it can be extracted by solving the coupled electromechanical field equation in layered media. As for the guiding structure of SiO_2_/36° YX LiTaO_3_, the optimal normalized SiO_2_ thickness to achieve lower TCF and maximum mass sensitivity is addressed by ~0.23 [17]. The corresponding COM parameters at optimum normal SiO_2_ thickness were extracted by FEM analysis mentioned above, as listed in Table 1.

**Figure 5:**
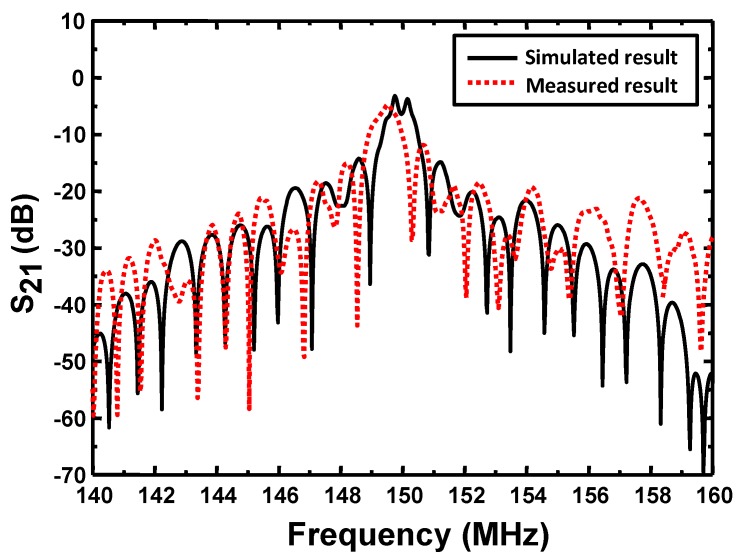
The simulated and measured response characteristics of the proposed Love wave device.

The following work is to simulate the Love wave sensing device by means of typical COM theory and the extracted COM parameters listed in Table 1 Usually, the frequency characteristic, S_12_, of Love wave device can be computed by following equation:

(4) $$S_{12}=\frac{-2Y_{12}\sqrt{Y_{01}Y_{02}}}{\left(Y_{01}+Y_{11}\right)\left(Y_{02}+Y_{22}\right)-Y_{12}Y_{21}}\;$$

where *Y*_11_, *Y*_12_, *Y*_21_ and *Y*_22_ are the elements of admittance matrix deduced by cascading the P-matrix of SPUDTs. *Y*_01_ and *Y*_02_ denotes the characteristic admittance of signal ports. Figure 5 shows the computed response characteristics (S_21_) of the Love wave devices with an operation frequency of 150 MHz. The corresponding device structure consists of two 300 nm Al-SPUDTs with lengths of 196λ (~26 μm) and 60λ. The acoustic aperture and guiding SiO_2_ overlay thickness are set to 100λ and 5 μm, respectively.

## 3. Technique Realization

### 3.1. Love Wave Devices

#### 3.1.1. SH-SAW Delay Line Preparation

As mentioned in Figure 1b, the proposed Love wave sensing device was composed of a SH-SAW delay line pattern on 36° YX LiTaO_3_ substrate with a SiO_2_ guiding layer, and a sensitive layer on top of the SiO_2_. First, a 150 MHz SH-SAW delay-line configuration was defined was fabricated photolithographically on a 36° YX LiTaO_3_ wafer. Two aluminum (Al) SPUDTs were separated by a path length of 2.5 mm. The corresponding wavelength, λ, is calculated though dividing the velocity by operation frequency as 26 μm. The electrode widths in SPUDTs are 6.5 μm (λ/4) and 3.25 μm (λ/8), respectively. The corresponding fabrication procedure is described below. Aluminum with thickness of 150 nm was deposited on the cleaned LiTaO_3_ substrate surface using a e-beam evaporation. Then, a 1-mm-thick photoresist (PR) was spin-coated, exposed, and developed for the delay line patterns. Al was wet-etched and PR was dissolved in acetone. Several rinses with DI water were performed to remove any unwanted products.

#### 3.1.2. SiO_2_ Guiding Layer Deposition

**Figure 6:**
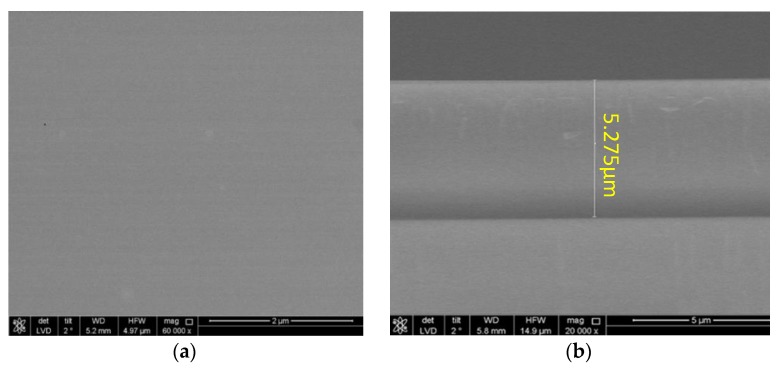
The AFM characterizing picture of the prepared SiO_2_ overlay, (**a**) surface characteristic; and (**b**) thickness measurement.

The SiO_2_ guiding layer with various thicknesses was deposited on the entire surface of the prepared patterned LiTaO_3_ wafer by ion-assisted e-beam evaporation. High-purity (99.99%) SiO_2_ target and vacuum degree of 10^−4^ Pa were utilized. The ion energy in ion-assist is adjusted to 120 eV, and the efficiency in evaporation is set to 1 nm/s. To prevent breakage of the piezoelectric substrate in the process of thicker SiO_2_ deposition over 200 °C, the e-beam evaporation was used for thicker SiO_2_ deposition at room temperature of 25 °C. The prepared SiO_2_ overlay with thickness of ~5 μm was characterized by AFM, the corresponding AFM picture indicates satisfactory quality of the SiO_2_ coating as good uniformity and less surface pollution, as shown in Figure 6.

#### 3.1.3. Love Wave Device Characterization

**Figure 7:**
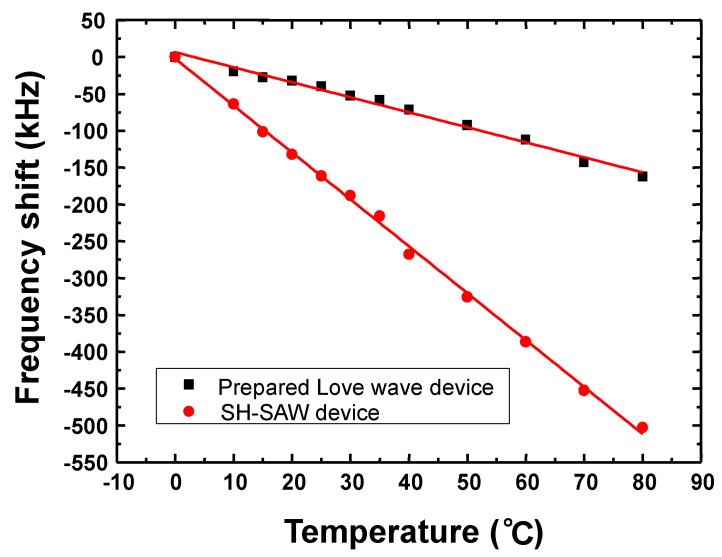
The measured temperature stability of the Love wave device.

The frequency response (S_21_) of the Love wave device was characterized by using the network analyzer as shown in Figure 6. Lower insertion of less than 5 dB was observed thanks to the waveguide effect and SPUDTs structure, which is in accordance with the theoretical prediction. Also, the temperature characteristic of the proposed Love wave device was performed by measuring its corresponding frequency response at various temperatures, as shown in Figure 7. It denotes that excellent temperature stability (TCF of 10 ppm/°C) was achieved by using the strategic guiding structure over the SH-SAW device on 36° YX LiTaO_3_ (39.8 ppm/°C).

### 3.2. Sensing Material Preparation

The CrypA employed for sensing CH_4_ was synthesized from vanilline by a so-called two-step method [11]. The CrypA solution was prepared prior to conducting the gas experiment. The polyvinyl chloride (PVC) and dioctyl sebacate (DLS) was used as the crosslinker to create covalent bonds among the CrypA molecules, while the tetrahydrofuran (THF) was utilized as the solvent. The detailed composition in CrypA solution is that 3.0 mg CrypA, 0.3 mg PVC and 0.6 mg DLS were dissolved in 2 mL THF. Prior to the CrypA deposition, the SiO_2_ surface of the sensing SAW device was cleaned by a routine cleaning procedure involving rinsing in piranha solution (H_2_SO_4_ − H_2_O_2_ = 3:1 *v*/*v*), a DI water rinse and drying by N_2_. Then, 0.3 μL CrypA solution was dropped on the cleaned SiO_2_ layer surface between the transducers of the sensing device, and then cured at 80 °C for 40 min in an oven.

**Figure 8:**
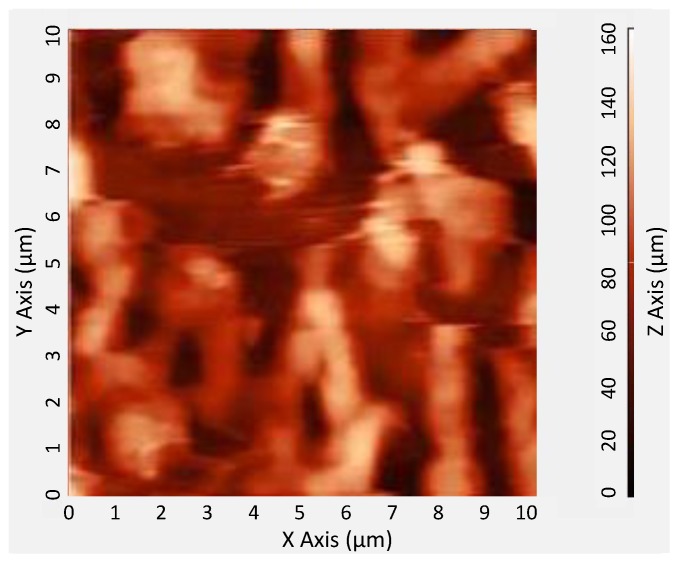
Surface topography description of the CrypA by AFM.

The surface topography of coated CrypA was characterized by the atomic force microscope (AFM), as shown in Figure 8. It is obviously that it has a rough surface with many fluctuations and bubbles, which is beneficial for gas sensing.

### 3.3. Differential Oscillator Configuration

**Figure 9:**
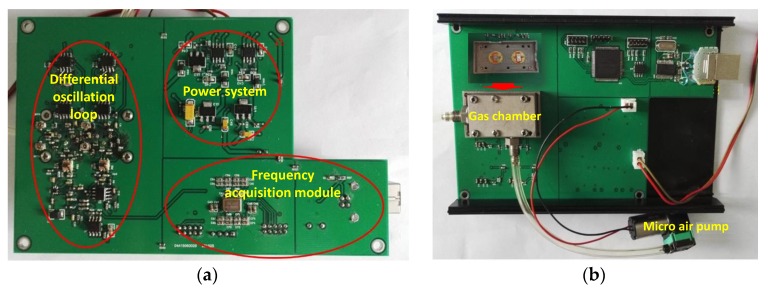
(**a**) The differential oscillation loop and (**b**) integrated sensor system.

To build the sensor system, the prepared CrypA coated Love wave device and a uncoated device as reference were connected into the differential oscillation loop depicted in Figure 9a, which was composed of amplifiers, phase shifters, and mixer. The differential frequency signal was picked by the FPGA based frequency acquisition module (FSM), and recorded and plotted by the PC. The prepared sensing devices were embedded into the nickel-plated Al-gas chamber with volume of 500 mL (Figure 9b). The gas in the air bags can be pumped into the gas chamber inside by an atmosphere sampler and a micro air pump. The corresponding PCB for the sensor system is shown in Figure 9.

**Figure 10:**
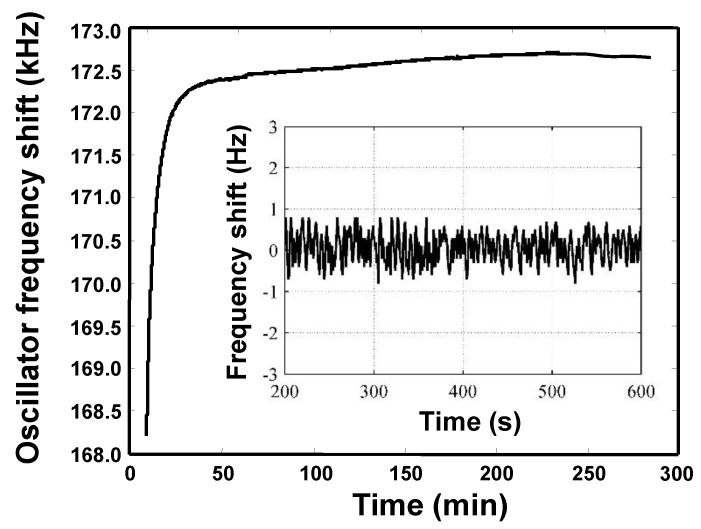
Frequency stability test of the proposed sensor system.

Obviously, the frequency stability of the oscillator affects directly the detection limit and stability of the sensor system, hence, a measurement on the frequency stability of the oscillator was conducted, as shown in Figure 10. After 20 min, the sensor reached a relatively stable state, and excellent short-term (in second) and medium-term (in hours) frequency stability were achieved as ±1 Hz/s and ±10 Hz/h.

## 4. Discussion Experimental Results and Discussions

**Figure 11:**
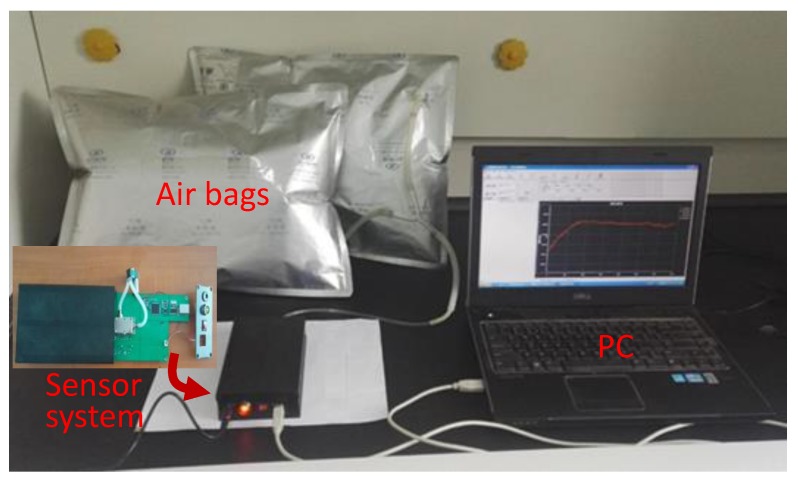
The experimental setup for characterizing the proposed Love wave sensor.

**Figure 12:**
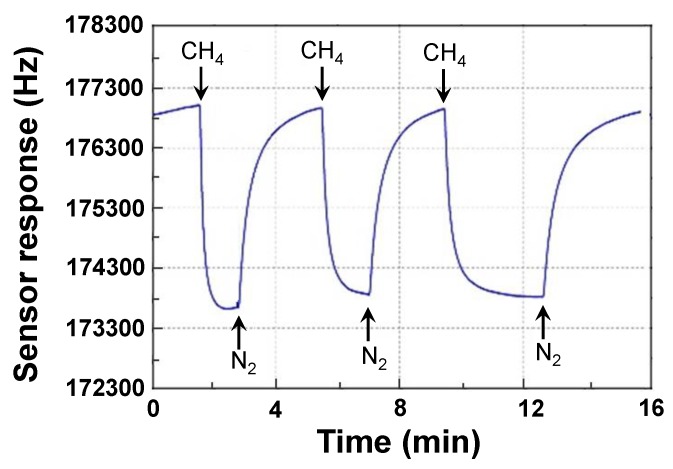
The repeatability test of the proposed sensor upon exposure to 5% CH_4._

Using the experimental setup composed of the proposed sensor system, air bags, hygrometer, thermometer, and PC, the Love wave sensing devices were characterized, as shown in Figure 11. First, the repeatability of the CrypA coated Love wave device was evaluated, as shown in Figure 12, that is a response profile obtained from the three consecutive 3 min on-off exposure to 5% CH_4_ in pure N_2_ at temperature/humidity of 25 °C/50% RH using the prepared Love wave sensing device. Excellent reproducible run was observed from the three gas exposures. Also, the picked sensor signal rises rapidly upon exposure to 5% CH_4_ and reaches the equilibrium value in 15 s, and then returns to its initial status within 20 s after removing the CH_4_. It means fast response time and recovery time with good repeatability were achieved from the developed Love wave sensor prototypes at room temperature.

**Figure 13:**
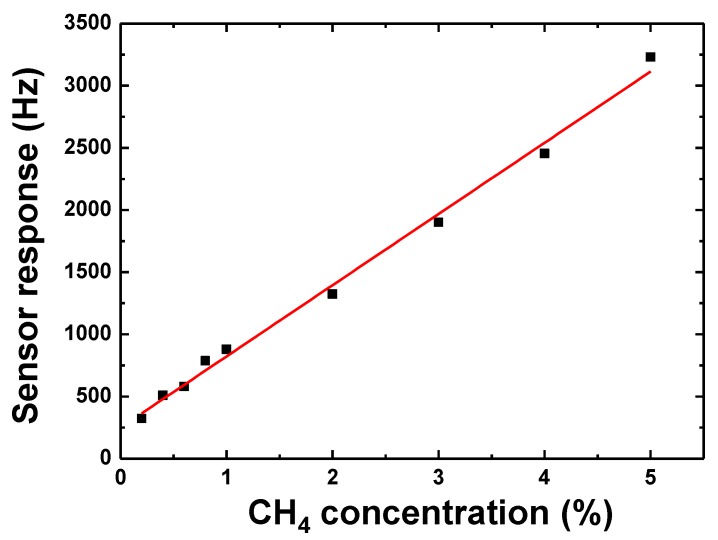
The sensitivity evaluation of the proposed Love wave sensor.

Figure 13 shows the sensor response at various CH_4_ concentration. It can be seen that the response is quite linear at CH_4_ concentrations of 0.1~5%, and the fitted slope is ~624 Hz/%, which is three times that of a similar sensor using R-SAW [10]. Also, a relatively high response of ~280 Hz is observed from the proposed Love wave sensing device upon exposure to 0.1% CH_4_, which means that a low detection limit of 0.005% is expected when a linear response was assumed at lower concentrations according to the International Union of Pure and Applied Chemistry (IUPAC) [6] norms.

**Figure 14:**
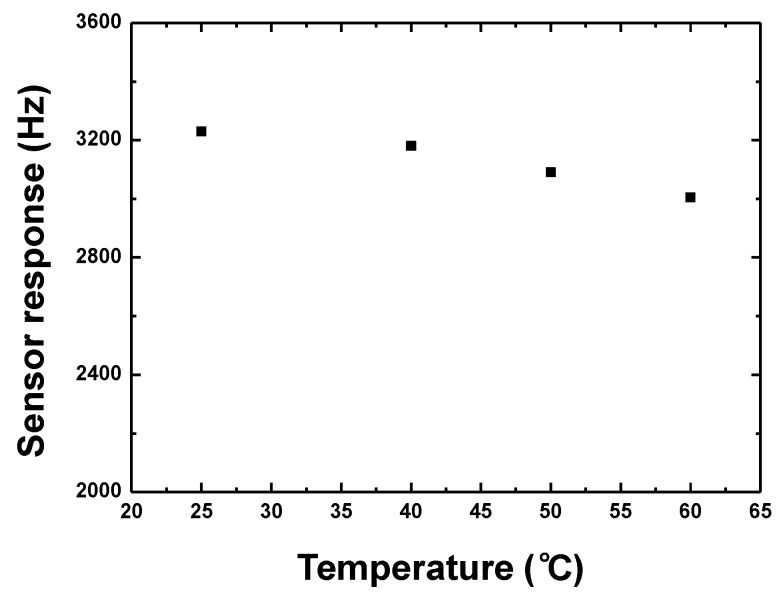
The temperature stability testing of the proposed sensor upon exposure to 5% CH_4_.

Also, the proposed sensor was characterized after exposure to 5% CH_4_ at various temperatures controlled by using a heating table to evaluate the temperature stability, as shown in Figure 14. Obviously, with increase of testing temperature, the sensor response towards 5% CH_4_ decreases. The response fluctuation is 6% from 25 °C to 60 °C, and only 0.2 %/°C thanks to the differential oscillation structure and self-compensated sensing device configuration. The reason for the response fluctuation arises from the non-zero Tcf of the Love wave device itself, CrypA thin-film and the active electronic components in the oscillation loop.

## 5. Conclusions

This work presents a new Love wave-based device for sensing methane gas, in which, a CrypA thin-film exhibits excellent sensor response, and the Love wave device provides a larger mass sensitivity and superior temperature stability. Using FEM analysis, the coupling of modes (COM) parameters were extracted for Love wave sensing device simulation prior to fabrication. The CrypA was synthesized by a typical two-step method, and coated onto the wave propagation path by using a dropping method. The developed Love wave sensing device was connected to a differential oscillation loop, and characterized in gas exposure experiments. High sensitivity, fast response, and excellent temperature stability were achieved at room temperature. Obviously, the proposed Love wave device features higher sensitivity compared to Rayleigh wave mode devices.

## Figures and Tables

**Table 1:** The COM parameters of the Love wave device at optimum SiO_2_ thickness of 0.23.

COM Parameters	Values	COM Parameters	Values
SAW velocity (m/s)	3962.08	Normalized excitation coefficient	0.0196
Normalized coupling coefficient (Ω^−1/2^)	4.7628 × 10^−4^	Normalized static capacitance (F/m)	4.6736 × 10^−10^
